# Uremic stomatitis: oral manifestations

**DOI:** 10.11604/pamj.2022.42.102.32685

**Published:** 2022-06-07

**Authors:** Sultan Meteb Alshammari, Rakhi Issrani

**Affiliations:** 1 College of Dentistry, Jouf University, Sakaka, Kingdom of Saudi Arabia,; 2 Department of Preventive Dentistry, College of Dentistry, Jouf University, Sakaka, Kingdom of Saudi Arabia

**Keywords:** Uremic stomatitis, mouth burning, blood urea nitrogen

## Image in clinical medicine

A 26-year-old male patient presented to Dental Clinic of Jouf University, Saudi Arabia with a chief complaint of white patches and burning sensation in his mouth for 2 weeks. Patient also reported with an accidental tongue biting that lead to a painful ulcer on the left lateral border of the tongue a few days before consultation. He had a history of vitamin D deficiency and renal weakness since 10 years back. History of drugs taken as allopurinol, omeprazole, prednisolone and antifungal drugs. History of present illness revealed difficulty while eating food and pain for two weeks. Extra-oral examination revealed no abnormality. Intra-oral examination showed white plaque-like lesions on the dorsal, lateral (A, B), ventral surfaces of tongue, buccal mucosa and on the lower labial mucosa (C). The lesions were present bilaterally on the tongue and buccal mucosa. Also, some wart-like projections were seen on the left buccal mucosa at the occlusal line (D). Further intra-oral examination revealed an ulcer measuring approximately 0.5x0.5 cm on the left lateral surface of the tongue (E). The ulcer was characterized of a reddish, well-defined border with slight elevation. The patient started orthodontic treatment using fixed braces. Laboratory investigation showed blood urea nitrogen abnormally high (BUN) as 200 mg/dl (normally; 9-20 mg/dl), creatinine level 18 mg/dl (normally range; 0.7-1.5 mg/dl). Pathological report was unavailable because the patient declined biopsy.

**Figure 1:**
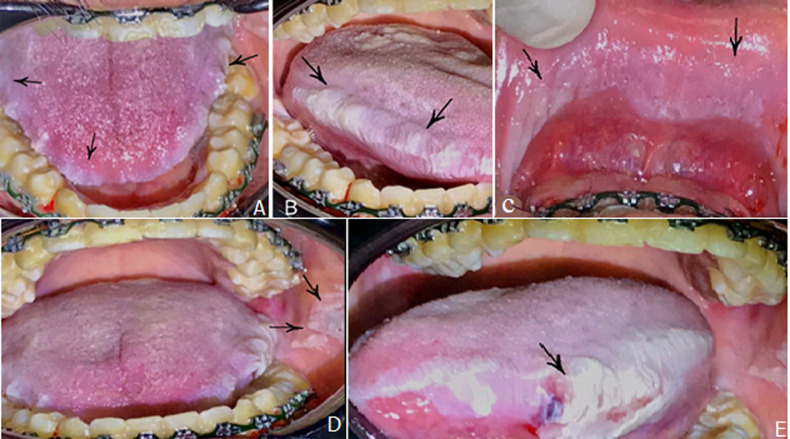
A,B) white plaque-like lesions on the dorsal, lateral, ventral surfaces of the tongue; C) buccal mucosa on the lower labial mucosa; D) wart-like projections on the left buccal mucosa at the occlusal line; E) ulcer on the left lateral surface of the tongue

